# The antiviral effects of RSV fusion inhibitor, MDT‐637, on clinical isolates, vs its achievable concentrations in the human respiratory tract and comparison to ribavirin

**DOI:** 10.1111/irv.12503

**Published:** 2017-10-30

**Authors:** Young‐In Kim, Rajat Pareek, Ryan Murphy, Lisa Harrison, Eric Farrell, Robert Cook, John DeVincenzo

**Affiliations:** ^1^ Department of Pediatrics University of Tennessee Health Science Center Memphis TN USA; ^2^ Children's Foundation Research Institute at Le Bonheur Children's Hospital Memphis TN USA; ^3^ College of Medicine University of Tennessee Health Science Center Memphis TN USA; ^4^ Teva Global Respiratory R&D Teva Pharmaceuticals Monmouth Junction NJ USA; ^5^ Department of Microbiology, Immunology, and Biochemistry University of Tennessee Health Science Center Memphis TN USA

**Keywords:** antiviral, bronchiolitis, fusion inhibitor, pharmacokinetics, respiratory syncytial virus, ribavirin

## Abstract

**Background:**

Respiratory syncytial virus (RSV) viral load and disease severity associate, and the timing of viral load and disease run in parallel. An antiviral must be broadly effective against the natural spectrum of RSV genotypes and must attain concentrations capable of inhibiting viral replication within the human respiratory tract.

**Objectives:**

We evaluated a novel RSV fusion inhibitor, MDT‐637, and compared it with ribavirin for therapeutic effect in vitro to identify relative therapeutic doses achievable in humans.

**Method:**

MDT‐637 and ribavirin were co‐incubated with RSV in HEp‐2 cells. Quantitative PCR assessed viral concentrations; 50% inhibitory concentrations (IC_50_) were compared to achievable human MDT‐637 and ribavirin peak and trough concentrations.

**Results and conclusions:**

The IC_50_ for MDT‐637 and ribavirin (against RSV‐A Long) was 1.42 and 16 973 ng/mL, respectively. The ratio of achievable peak respiratory secretion concentration to IC_50_ was 6041‐fold for MDT‐637 and 25‐fold for aerosolized ribavirin. The ratio of trough concentration to IC_50_ was 1481‐fold for MDT‐637 and 3.29‐fold for aerosolized ribavirin. Maximal peak and trough levels of oral or intravenous ribavirin were significantly lower than their IC_50_s. We also measured MDT‐637 IC_50_s in 3 lab strains and 4 clinical strains. The IC_50_s ranged from 0.36 to 3.4 ng/mL. Achievable human MDT‐637 concentrations in respiratory secretions exceed the IC_50_s by factors from hundreds to thousands of times greater than does ribavirin. Furthermore, MDT‐637 has broad in vitro antiviral activity on clinical strains of different RSV genotypes and clades. Together, these data imply that MDT‐637 may produce a superior clinical effect compared to ribavirin on natural RSV infections.

## 1 INTRODUCTION

Respiratory syncytial virus (RSV) causes acute bronchiolitis and pneumonia, which are important causes of childhood illness and death. Every year ~3% of all US infants are hospitalized with RSV infection most of whom are previously healthy.^1^ The burden of RSV in outpatients is even greater with ~39% of all outpatient visits under 2 years of age caused by this virus.^2^ Treatment with nebulized beta‐2 agonists, epinephrine, Heliox, or surfactant has been studied. But, the trials have either not shown convincing clinical benefit or offer very short‐term benefit, which does not alter the underlying disease process.^3^, ^4^, ^5^, ^6^, ^7^ Corticosteroids have not shown benefit^8^ and increase the concentration of RSV in the respiratory tract.^9^ Nebulized hypertonic saline has also not shown benefit.^10^ Ribavirin inhibits RSV replication and is the only FDA‐approved specific treatment for RSV lower respiratory tract infection. Aerosolized ribavirin has been demonstrated to achieve around one log reduction in RSV‐infected cotton rat lungs and reduces RSV measured from children's nasal secretions.^11^, ^12^ All randomized trials of aerosolized ribavirin therapy in children have shown either a statistically significant clinical benefit or a trend in this direction; however, the treatment and antiviral effects are small.^13^ After 3 days of aerosolized ribavirin therapy, hospitalized children showed a reduction of 0.6 log pfu/mL compared to controls.^13^ This ribavirin small antiviral effect is controversial because the method of RSV quantification employed in the studies utilized RSV quantitative culture, which is known to be affected ex vivo by concentrations of ribavirin within the secretions themselves. Therefore, the ribavirin data suggest that inhibiting RSV replication might reduce disease severity in children, especially when an antiviral is more potent than ribavirin. Ribavirin aerosol can only be delivered practically to inpatients because of its teratogenicity, the environmental contamination issues, and concern for healthcare worker exposure during treatment, necessitating the elaborate use of environmental protection devices. Additionally, ribavirin aerosol must dose either continuously or for three, dosing times each day, each lasting 2 hours. Intravenous and oral ribavirin have never been adequately studied for RSV therapy. No licensed vaccines currently exist for preventing RSV infections. Prophylactic administration of monoclonal antibodies has been effective in reducing, but not eliminating, severe RSV disease, and this antibody is only recommended for under 3% of the birth cohort in the United States.^14^ Therapeutic uses of high potency RSV‐monoclonal antibody have not shown clinical benefit.^15^, ^16^, ^17^ The pediatric population needs an efficient and easily applied therapy for RSV.

More potent RSV antivirals are promising because higher viral loads and greater disease severity correlate in infants and more rapid natural clearance of RSV and shorter hospital stays correlate.^18^, ^19^ Furthermore, we have recently shown that the timing of RSV load and disease track together.^20^ This implies that reducing viral load may translate into clinical benefit. Even at a time when viral replication is at its highest, application of antiviral agents early in the disease course might improve subsequent morbidity by significantly lowering viral load, reducing direct viral cytopathic effects, and aborting potential downstream immunopathology.^19^, ^21^, ^22^

A major consideration for candidate experimental RSV antivirals is the requirement that the molecule is broadly active across the natural spectrum of viral genotypes.^23^ The RSV G protein, which mediates viral attachment to target cells, is the most genetically and antigenically diverse RSV protein and forms the basis of genotyping. We have retained a series of primary low‐passage isolates from a broad clade range including genotypes RSV‐A and RSV‐B (Fig. S1).^23^ Antiviral activity of any new therapeutic must be preserved across this wide range of diverse RSV isolates.

MDT‐637 (previously called VP14637, Teva Pharmaceuticals, Tel Aviv, Israel) is a substituted bis‐tetrazole‐benzhydryl phenol, which has been reported to reduce RSV replication by inhibition of the F‐protein function.^24^ This molecule has produced an antiviral effect in vivo (cotton rats) following delivery by small droplet aerosol.^25^ It has been reformulated so as to be deliverable to humans. A phase‐1 evaluation of single and multiple ascending doses of aerosolized MDT‐637 to determine its safety and pharmacokinetics has been completed (ClinicalTrials.gov identifier NCT01475305).

We therefore evaluated the in vitro antiviral effects of MDT‐637 (50% inhibitory concentration, IC_50_) against a broad range of genetically diverse RSV strains and compared it to ribavirin. We also related the IC_50_ of MDT‐637 and ribavirin to the concentrations achieved within human respiratory secretions so as to evaluate the relative antiviral effect in humans infected with community‐acquired RSV.

## 2 MATERIALS AND METHODS

### 2.1 Cells and viruses

Human epithelial carcinoma (HEp‐2) cells (ATCC, CCL‐23) were used for RSV replication in the presence of MDT‐637 or ribavirin. RSV‐A Long (ATCC, VR‐26) and A2 (ATCC, VR‐1540) and RSV‐9320 (ATCC, VR‐955) were maintained by serial passage in the laboratory. A broad range of RSV clinical isolates (RSV‐Memphis‐37, RSV‐LAP0824, RSV‐HAN1135; Fig. S1) representative of the major clades of RSV‐A and RSV‐B was selected for this study. This study was conducted with the approval of the Institutional Review Board of the University of Tennessee Health Science Center.

### 2.2 Compounds

MDT‐637 (MicroDose Therapeutx Inc., NJ, USA) was solubilized in 5 mL of 100% dimethylsulfoxide. Consecutive 10‐fold serial dilutions subsequently were prepared with DMEM until the first experimental drug concentration (0.1 μmol/L) was reached. Half‐log dilutions were then performed with DMEM from 0.1 μmol/L to 31.6 pmol/L. Ribavirin (Calbiochem, CA, USA) was diluted using the same serial dilution as described for MDT‐637.

### 2.3 Exposure of RSV to Drug

The laboratory and clinical isolates of RSV (Multiplicity of Infection [MOI]; 0.001) and various concentrations (ranging from 0.1 μmol/L to 31.6 pmol/L) of MDT‐637 (or ribavirin) were added to the HEp‐2 cell monolayers at the same time. After 72 hours of incubation at 37°C, the cells and supernatants were harvested. An aliquot was immediately serially diluted and used for plaque assay as described in section “*Quantification by Culture*.” The remainder cell‐supernatant mixture was frozen at −80°C and subsequently quantified by qPCR.

### 2.4 Quantification by culture (qCx)

Plaque assays were performed as previously described^26^ using the human RSV‐A Long strain (for A strains) and RSV‐B, 9320 (for B strains) as a quantitative standard.

### 2.5 Quantitative real time‐PCR (qPCR)

Viral RNA was extracted from the samples frozen at −80°C using QIAsymphony Virus/Bacteria Mini/Midi Kit (QIAGEN, Valencia, CA, USA) according to the manufacturer's instructions and incubated with DNA‐free DNase (TURBO DNA‐free Kit, Applied Biosystems, Foster City, CA). The viral RNA extraction used the QIAsymphony Pathogen Complex 400 Protocol on the QIAsymphony^SP^ Viral Extractor Robot (Serial No. 10502). Following extraction, cDNA was obtained through reverse transcription using the QIAGEN Omniscript RT Kit (QIAGEN, Valencia) and N gene‐specific custom primers (Applied Biosystems, Foster City, CA)^26^. PCR was performed as previously described.^27^

### 2.6 Statistics

All statistical analyses were performed using graphpad prism, Version 5.0d.

## 3 RESULTS

### 3.1 Comparison of the in vitro antiviral effect of MDT‐637 to ribavirin

**Figure 1:**
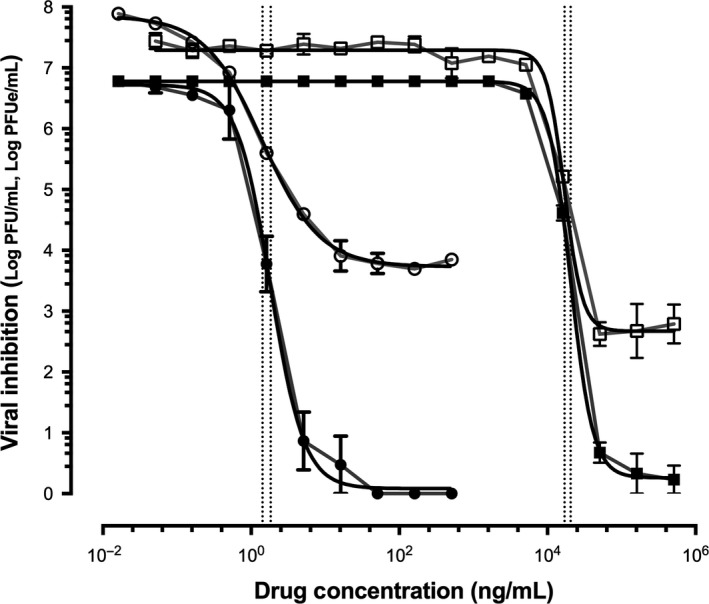
Inhibition of Respiratory syncytial virus (RSV)‐A Long by MDT‐637 (represented by closed and open circles) and ribavirin (represented by closed and open squares) measured by quantitative culture (represented by closed circles and closed squares, quantification unit; log plaque forming units/mL, log PFU/mL) and quantitative PCR (represented by open circles and open squares, quantification unit; log plaque forming unit equivalents/mL, log PFUe/mL). The vertical dotted lines represent the IC_50_ for MDT‐637 (1.83 ng/mL by quantitative culture and 1.42 ng/mL by quantitative PCR) and ribavirin (20 509 ng/mL by quantitative culture and 16 973 ng/mL by quantitative PCR)

The IC_50_s for MDT‐637 and ribavirin against RSV‐A Long were 1.42 ng/mL and 16 973 ng/mL by qPCR, respectively. By qCulture, the IC_50_s were 1.83 ng/mL for MDT‐637 and 20 509 ng/mL for ribavirin. These represent over 11 000‐fold greater potency of MDT‐637 (Table 1 and Figure 1).

**Table 1:** Comparison of IC_50_s for MDT‐637 and ribavirin within Respiratory syncytial virus (RSV)‐A Long by quantitative culture and quantitative PCR

	IC_50_ MDT‐637 (ng/mL)	IC_50_ Ribavirin (ng/mL)
qPCR (95% CI^a^)	1.42(1.2‐1.68)	16 973(15 155‐19 010)
qCx (95% CI)^b^	1.83(1.53‐2.18)	20 509( )(19 515‐21 554)

a CI, Confidence Interval.

b CI is calculated based on the experiments performed in duplicate and with each experiment having two separate qPCR determinations per experiment per drug concentration.

### 3.2 In vitro antiviral effect of MDT‐637 against broad range of RSV isolates

MDT‐637 IC_50_s were measured with three laboratory strains (RSV‐A Long, RSV‐A2, and RSV‐9320) and three clinical strains (RSV‐Memphis‐37, RSV‐LAP0824, and RSV‐HAN1135; Fig. S1). The IC_50_s ranged from 0.36 to 3.4 ng/mL measured by qPCR (Table 2). IC_50_s were measured by qCx for RSV strains, A Long, A2, and Memphis‐37, and correlated with the corresponding IC_50_s measured by qPCR validating the fidelity of the two measurement techniques (Table S1). For subsequent strains, antiviral effect was measured only by the qPCR.

**Table 2:** MDT‐637 IC_50_s within various laboratory and clinical Respiratory syncytial virus (RSV) strains representing broad genotypic diversity

RSV genotype	RSV strain (clade name)	Source	MDT‐637 IC_50_(ng/mL)	95% CI^a^^,^ b
A	A Long (NH/A1)	Laboratory	1.42	1.2‐1.68
	A2 (NH/A1)	Laboratory	3.4	2.25‐5.13
	Memphis‐37 (NH/A2)	Clinical	1.4	0.98‐1.98
	LAP0824 (NH/A4)	Clinical	0.36	0.30‐0.43
B	9320 (NH/B1)	Laboratory	1.73	1.16‐2.58
	HAN1135 (NH/B3)	Clinical	0.61	0.35‐1.06

a CI, Confidence Interval.

b CI is calculated based on the experiments performed in duplicate and with each experiment having two separate qPCR determinations per experiment per drug concentration.

### 3.3 MDT‐637 concentrations in human respiratory secretions compared to IC_50_s

MDT‐637 has poor aqueous solubility and approximately 180‐fold greater solubility in simulated lung fluid.^28^, ^29^, ^30^ In a phase I clinical trial (ClinicalTrials.gov identifier NCT01475305), after safely receiving aerosolized MDT‐637, concentrations in saline nasal washes were measured, then extrapolated using this solubility factor and compared to the MDT‐637 IC_50_s obtained for RSV‐A Long and RSV‐Memphis‐37. Peak extrapolated MDT‐637 concentrations were over 6,000‐fold greater than the IC_50_s (Table 3).

**Table 3:** The ratios of MDT‐637 concentration in respiratory secretion to IC_50_s

Dosing time point	Concentration of MDT‐637 in respiratory secretion/IC_50_			
	From saline nasal wash, uncorrected for solubility		Using 1/180 aqueous to simulated lung fluid^29^, ^30^solubility ratio	
	RSV‐Memphis‐37	RSV‐A Long	RSV‐Memphis‐37	RSV‐A Long
0.25 hour peak	34.04‐fold^a^	33.56‐fold^a^	6128‐fold^a^	6041‐fold^a^
6 hour trough	8.34‐fold^a^	8.23‐fold^a^	1502‐fold^a^	1481‐fold^a^

a Mean of achievable nasal wash concentrations is from phase I clinical trial data of 66 μg aerosol TID and 132 μg aerosol TID. Peak and trough concentrations from the both dosing groups were measured pre‐dosing and 15 minutes post‐aerosol dosing on the 6th day of treatment and then averaged the values.

### 3.4 Ratios of achievable concentrations of RSV antivirals to their corresponding the IC_50_s

Using RSV‐A Long, the ratio of achievable peak respiratory secretion concentration to IC_50_ was 6,041‐fold for MDT‐637 and 25‐fold for ribavirin. The ratio of trough concentration to IC_50_ was 1,481‐fold for MDT‐637 and 3.29‐fold for ribavirin (Table 4).

**Table 4:** Ratios of achievable concentrations of Respiratory syncytial virus (RSV) antivirals to their corresponding IC_50_s

Antiviral	Route of administration	Fluid measured	Ratio of antiviral concentration (mg/dL) to IC_50_ (ng/mL)	
			Peak	Trough
MDT‐637^a^	Aerosol	Respiratory secretion	6041‐fold a ^,^ b	1481‐fold^a^^,^ b
Ribavirin	Aerosol	Respiratory secretion	25‐fold^35^	3.29‐fold^35^
Ribavirin	Oral (single‐dose)	Plasma	0.05‐fold^38^, ^39^, ^40^	‐
Ribavirin	Oral (multiple‐dose)	Plasma	0.22‐fold^38^, ^39^, ^40^	0.13‐fold^35^
Ribavirin	Intravenous (single‐dose)	Plasma	0.25‐fold^41^	0.004‐fold^41^

a 1/180 aqueous to simulated lung fluid solubility ratio is used in the calculations.

b Mean of achievable nasal wash concentrations are from phase I clinical trial data of 66 μg aerosol TID and 132 μg aerosol TID. Peak and trough concentrations from the both dosing groups were measured pre‐dosing and 15 minutes post‐aerosol dosing on the 6th day of treatment and then averaged the values.

## 4 DISCUSSION

Treatment options for RSV are suboptimal. In search of effective antivirals, the RSV F‐protein has emerged as an active target. Multiple novel small compounds acting as RSV entry inhibitors by targeting the F protein have been studied. Many of them have demonstrated potent in vitro antiviral effects. We have completed preclinical and early clinical studies for several of these compounds.^31^, ^32^

Ribavirin, a nucleoside analogue (anti‐RSV small molecule), is the only FDA‐approved RSV therapeutic. But, it has questionable and limited antiviral efficacy and almost no clinical use in current times. However, it offers a standard to which new therapeutics can be compared. Previous antiviral effect comparison studies have been performed between ribavirin and MDT‐637. Douglas et al have demonstrated the in vitro antiviral effects of MDT‐637 and ribavirin using cytopathic effect‐inhibitory assay.^24^ Their cytopathic effect assay showed a 50% effective concentration (EC_50_) for MDT‐637 was 1.4 nmol/L (or 0.715 ng/mL), whereas the EC_50_ for ribavirin was 33 000 nmol/L (or 16 847 ng/mL) against the RSV‐A2 strain. Wyde et al demonstrated the antiviral effect of MDT‐637 and ribavirin against RSV‐A Long and the EC_50_s were 0.001 ± 0.004 μmol/L (or 0.51 ± 2.04 ng/mL) and 20 ± 10 μmol/L (or 10,210 ± 5,105 ng/mL), respectively.^25^ These studies demonstrated MDT‐637 was over 20 000‐fold more potent than ribavirin. In the present study, we have shown IC_50_s for MDT‐637 against RSV‐A Long and RSV‐A2 were 1.83 ng/mL and 2.708 ng/mL, respectively (by qCx), and 1.42 ng/mL and 3.4 ng/mL, respectively (by qPCR). We also compared the in vitro antiviral effects of MDT‐637 to ribavirin against RSV‐A Long strain. In line with previously reported data, our data support that MDT‐637 is over 11 000‐fold more potent than ribavirin in vitro (IC_50_ ribavirin 20 509 ng/mL [qCx] and 16 973 ng/mL [qPCR]). Our findings are in line with previously reported data.

We extended these findings to evaluate activity across a series of RSV low passage, strains representing a broad range of genotypic diversity. Our clinical isolates represented the major clades of both RSV‐A and RSV‐B to show that MDT‐637 retains antiviral effects against the wide range of RSV genotypes. The IC_50_s of MDT‐637 ranged from 0.36 to 3.4 ng/mL (qPCR). These strain‐specific differences are small and are unlikely to affect the activity of MDT‐637 given the high achievable respiratory tract concentration. Similar RSV strain differences in fusion inhibitor activity have been previously reported.^33^, ^34^

The extent to which the achievable human concentration of an antiviral exceeds its in vitro inhibitory level can predict its clinical efficacy. We therefore related the IC_50_s to the concentration of MDT‐637 and ribavirin achieved in human respiratory secretions. Pharmacokinetic and pharmacodynamics studies of MDT‐637 in healthy human volunteers measured the mean drug concentrations in the nasal saline washes at 0.25 hours (peak) and 6 hours (trough) after exposure with 66 μg or 132 μg TID. The mean peak and trough concentrations were 47.66 ng/mL and 11.68 ng/mL, respectively. The mean peak and trough coefficient of variation were 98.40% and 157.32%, respectively. However, MDT‐637 has poor aqueous solubility; it is about 1/180th that of its solubility in a simulated lung fluid. MDT‐637 solubility in simulated lung fluid is approximately 1077 nmol/L, and the solubility in water is approximately 6 nmol/L.^30^ This solubility factor may be lower under working conditions. Using this solubility factor of 180, we calculated the MDT‐637 human respiratory secretion to in vitro MDT‐637 IC_50_ concentration ratios. Peak extrapolated MDT‐637 concentrations were over 6000‐fold greater than the IC_50_s. Moreover, the 6‐hour trough extrapolated concentrations were around 1500‐fold higher than the IC_50_s. We further compared these data with the achievable ribavirin concentrations in human respiratory secretions after aerosolized ribavirin administration. This information was based on the only publication measuring respiratory secretion concentrations of ribavirin in children during dosing of the drug in the format that is commonly used, that is*,* high dose, short course, three times a day dosing.^35^ Using RSV‐A Long, the ratio of achievable peak respiratory secretion concentration to IC_50_ was 6041‐fold for MDT‐637 and 25‐fold for aerosolized ribavirin. The ratio of trough concentration to IC_50_ was 1481‐fold for MDT‐637 and 3.29‐fold for aerosolized ribavirin (Table 4). Under the working condition, achievable MDT‐637 concentrations in respiratory secretions exceed the IC_50_s by factors from several hundred‐ to thousand‐fold greater than does aerosolized ribavirin concentrations in human respiratory secretions. The commonly reported clinical practice of administering ribavirin by the oral and intravenous route for severe RSV infection was also evaluated.^36^, ^37^ Our results predict the futility of such oral or intravenous ribavirin dosing for this infection.

## 5 CONCLUSIONS

This study looking at the antiviral effects of the RSV F‐protein inhibitor MDT‐637 shows that MDT‐637 appears to have antiviral activity against laboratory and clinical strains representing broad RSV genetic diversity. It is from several hundred‐ to thousand‐fold more potent in vitro compared to ribavirin. MDT‐637 concentrations achievable in human nasal washes are from hundred‐ to thousand‐fold higher than the IC_50_s, suggesting that an antiviral effect may be achievable in human. Comparing IC_50_s to achievable human drug concentrations predicts that MDT‐637 may produce a superior clinical effect compared to ribavirin on natural human RSV infections.
