# Supplementary figures and images for: The antiviral effects of RSV fusion inhibitor, MDT‐637, on clinical isolates, vs its achievable concentrations in the human respiratory tract and comparison to ribavirin
Source: Influenza Other Respir Viruses. 2017 Oct 30;11(6):525–30. doi: 10.1111/irv.12503 (PMC5705693; doi:10.1111/irv.12503)

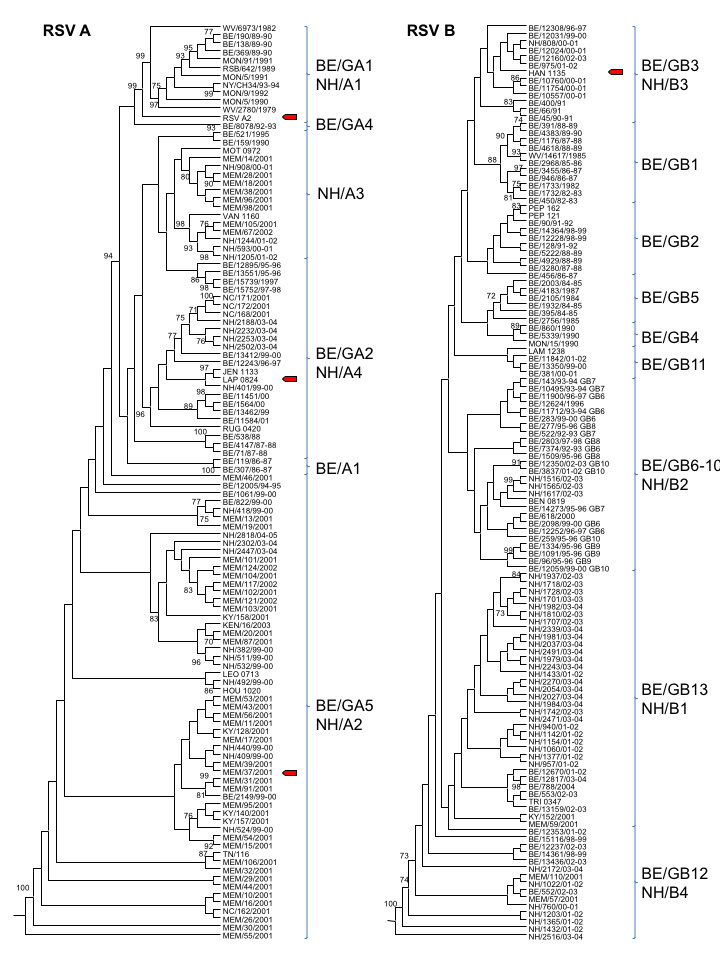

Supplement: Supplementary file 1 [file IRV-11-525-s001.tiff]
