# Supplementary material for: The antiviral effects of RSV fusion inhibitor, MDT‐637, on clinical isolates, vs its achievable concentrations in the human respiratory tract and comparison to ribavirin
Source: Influenza Other Respir Viruses. 2017 Oct 30;11(6):525–30. doi: 10.1111/irv.12503 (PMC5705693; doi:10.1111/irv.12503)
Supplement: Supplementary file 2 [file IRV-11-525-s002.docx]

**Supplementary Table 1.**

**MDT-637 IC_50_s measured by quantitative Culture within various laboratory RSV strains.**

| **RSV Genotype** | **RSV Strain (Clade Name)** | **Strain Source** | **MDT-637 IC_50_*^a^*(ng/ml)** | **95% CI*^b,c^*** |
| --- | --- | --- | --- | --- |
| **A** | A Long(NH/A1) | Laboratory | 1.83 | 1.5-2.18 |
|  | A2 (NH/A1) | Laboratory | 2.7 | 2.2-3.33 |
|  | Memphis-37 (NH/A2) | Clinical | 1.2 | 0.95-1.57 |

^a^IC_50_, 50% Inhibitory Concentration

^b^CI, Confidence Interval

^c^The Confidence Interval is calculated based on the experiments performed in duplicate and with each experiment having two separate qPCR determinations per experiment per drug concentration.
